# Comparative Analysis of Fatty Acid Composition in Skeletal Muscles and Serum of Tianfu Goats

**DOI:** 10.3390/foods15203586

**Published:** 2026-10-09

**Authors:** Xiaohui Meng, Meijun Song, Dinghui Dai, Xiaoli Xu, Jiaxue Cao, Jiazhong Guo, Linjie Wang, Tao Zhong, Siyuan Zhan, Li Li, Hongping Zhang

**Affiliations:** 1 Key Laboratory of Livestock and Poultry Multi-Omics, Ministry of Agriculture and Rural Affairs, College of Animal Science and Technology, Sichuan Agricultural University, Chengdu 611130, China; 2 Farm Animal Genetic Resources Exploration and Innovation Key Laboratory of Sichuan Province, Sichuan Agricultural University, Chengdu 611130, China

**Keywords:** lipid profiling, meat-type goat, serum fatty acids, skeletal muscle

## Abstract

Fatty acid composition contributes to the nutritional characteristics of goat meat, but its variation among anatomical muscles remains insufficiently characterized. This exploratory study determined fatty acid profiles in six skeletal muscles and serum from nine 10-month-old castrated male Tianfu goats raised under a common farm feeding and management regimen using targeted gas chromatography–tandem mass spectrometry (GC–MS/MS). Oil Red O staining was also performed for semiquantitative histological analysis of the six skeletal muscles. Thirty-four fatty acids were detected in skeletal muscle, with oleic acid (C18:1*n*9c), palmitic acid (C16:0), stearic acid (C18:0), and linoleic acid (C18:2*n*6c) as the predominant components. Monounsaturated fatty acids (MUFAs) constituted the largest fatty acid subclass in skeletal muscle, whereas saturated fatty acids (SFAs) predominated in serum. The *longissimus dorsi* (LD) and *supraspinatus* (SS) showed relatively stronger Oil Red O-positive staining. The total fatty acid concentration was significantly higher in SS than in the *semimembranosus* (SM) muscle (18,282.09 ± 8516.92 vs. 5831.66 ± 3310.31 μg/g, *p* < 0.05). SS also contained higher concentrations of polyunsaturated fatty acids (PUFAs), SFAs, and MUFAs than SM (1065.99 ± 425.52 vs. 421.00 ± 103.96, 6973.64 ± 3325.02 vs. 2374.02 ± 1383.12, and 10,242.46 ± 4812.55 vs. 3036.65 ± 1841.48 μg/g, respectively; *p* < 0.05). Twenty-seven fatty acids were shared between serum and skeletal muscle, and correlation analysis with false discovery rate (FDR) correction revealed exploratory muscle-specific serum–muscle association patterns. Overall, Tianfu goat skeletal muscles exhibited a shared MUFA-dominant profile but clear anatomical variation in fatty acid abundance, with SS and LD showing potential for nutritional differentiation and value-added utilization.

## 1. Introduction

Goat meat is a lean red meat with substantial protein content and favorable nutritional characteristics [1,2,3]. Goat meat is generally regarded as a lean red meat and often exhibits a relatively favorable PUFA/SFA ratio compared with beef and pork [4,5,6]. These characteristics are consistent with healthy dietary recommendations that encourage the consumption of lean meats and the replacement of saturated fats with unsaturated fats [7]. In goats, fatty acid composition is influenced by multiple factors, including breed, diet, age, sex, anatomical muscle location, and lipid deposition status [8,9,10]. Therefore, characterizing fatty acid composition in goat skeletal muscles is important for understanding meat quality traits and for providing basic information for meat quality evaluation [6].

Anatomical muscles differ in contractile function, muscle fiber composition, metabolic characteristics, and lipid deposition, which may contribute to muscle-specific fatty acid profiles. Intermuscular variation in total lipid content has been well documented [11,12]. Because muscle phospholipids are relatively enriched in PUFAs, whereas neutral lipids generally contain higher proportions of SFAs and MUFAs, differences in total lipid content and lipid-class distribution may partly account for variation in fatty acid composition among muscles [11,13]. In Korean native black goats, the LD, *psoas major* (PM), SM and *gluteus medius* (GM) muscles exhibited distinct muscle fiber characteristics, fat contents, and fatty acid compositions [14]. Muscle-dependent fatty acid profiles have also been observed in other goat populations [15,16]. Accordingly, analysis of multiple anatomical muscles is necessary to characterize intermuscular variation, rather than relying on a single muscle to characterize the fatty acid composition of the whole carcass.

Serum fatty acid composition reflects the circulating fatty acid status of the animal and can provide a complementary reference for interpreting muscle fatty acid profiles [17]. Tracer and arteriovenous studies in sheep have demonstrated that skeletal muscle utilizes circulating non-esterified fatty acids [18,19]. In goats, major fatty acids, including C16:0, C18:0, oleic acid (C18:1*n*9) and linoleic acid (C18:2*n*6), have been detected in both serum and skeletal muscle, although their relative distributions differ between serum and among anatomical muscles [20]. Dietary *n*-3 fatty acid supplementation has also been shown to alter the fatty acid composition of different goat muscles to varying degrees [21]. Together, these findings indicate that serum fatty acid profiles can provide a useful physiological context for interpreting intermuscular variation in fatty acid composition.

Against this background, the Tianfu goat is a newly developed meat-type breed in China and represents an important genetic resource for goat meat production. However, systematic information on intermuscular variation in its fatty acid composition remains limited. Based on differences in muscle function and lipid deposition, this exploratory study examined the hypothesis that fatty acid concentrations and relative composition vary among anatomically distinct muscles within the same animal. To examine this hypothesis, fatty acid profiles were characterized in six skeletal muscles and serum of Tianfu goats raised under a common farm feeding and management regimen. The objectives were to determine intermuscular variation in fatty acid concentrations and composition and compare the distribution patterns of fatty acid species and subclasses between skeletal muscle and serum. Associations between serum and muscle fatty acid profiles were evaluated as an exploratory secondary objective. These analyses aimed to provide basic compositional data for the nutritional evaluation of Tianfu goat meat.

## 2. Materials and Methods

### 2.1. Animals and Sample Collection

Muscle and blood samples were collected from nine unrelated castrated male Tianfu goats at 10 months (±10 d) of age, with a mean slaughter weight of 45.2 ± 6.3 kg. One sample from each of the six anatomical muscles was collected from every goat, and the goat was considered the experimental unit in a paired within-animal design. This exploratory design enabled direct comparisons among anatomically distinct muscles within the same animal, thereby reducing between-animal variation and maximizing the information obtained from each experimental unit. By obtaining all six muscle samples from each goat, the design also supported the Reduction principle of the 3Rs (Replacement, Reduction, and Refinement). All goats were raised on the farm at the Ya’an Campus of Sichuan Agricultural University (Ya’an City, Sichuan Province) under a common farm feeding and management regimen. Animals were group-housed under natural temperature and ventilation conditions and shared feed troughs. They had free access to clean drinking water. Following weaning at 2 months of age, all goats remained under routine farm management until slaughter at 10 months of age. Feeding management was conducted with reference to the nutrient requirements for meat-type goats specified in the Chinese agricultural industry standard NY/T 816-2021, Nutrient Requirements of Meat-Type Sheep and Goats [22]. The basal diet consisted of oat hay, broad bean hulls, and concentrate, which were offered twice daily at 06:00 and 16:00 with ad libitum access. The feeds were offered sequentially, with concentrate provided first, followed by broad bean hulls and then oat hay. The concentrate was a commercial feed product comprising corn, wheat bran, corn germ meal, soybean meal, and sodium bicarbonate. No supplemental lipid source was included in the diet. The nutrient composition of the feed ingredients is presented in Table 1. Individual voluntary feed intake was not recorded. Therefore, equivalent nutrient intake among animals could not be verified.

Blood samples were collected from the jugular vein of each goat before slaughter into two 5 mL additive-free vacuum tubes. After allowing the blood to clot at 4 °C, the samples were centrifuged at 3000 r/min for 10 min at 4 °C using a Sorvall ST 8R centrifuge (Thermo Fisher Scientific, Waltham, MA, USA). The separated serum was transferred into sterile tubes and stored at −20 °C until targeted fatty acid analysis. Within 1 h after humane slaughter, samples of the SS, *infraspinatus* (IS), LD, PM, *semitendinosus* (ST), and SM muscles were collected. Muscle samples were immediately frozen in liquid nitrogen and stored at −80 °C until GC–MS/MS analysis. An additional muscle sample of approximately 1 cm^3^ was embedded in optimal cutting temperature (OCT) compound (catalog no. G6059; Servicebio, Wuhan, China), rapidly frozen in liquid nitrogen, and stored at −80 °C until cryosectioning for Oil Red O staining. All sampling procedures were reviewed and approved by the Animal Care and Use Committee of Sichuan Agricultural University (License Number: DKY-2021202036).

### 2.2. Oil Red O Staining and Image Quantification

Oil Red O staining was performed on frozen skeletal muscle sections according to previously described protocols, with minor modifications [23,24]. Frozen sections from the six skeletal muscles were used for histological evaluation. Muscle samples were embedded in OCT compound immediately after collection and stored at −80 °C until sectioning. The OCT-embedded samples were sectioned at a thickness of 8–10 μm using a CryoStar NX50 cryostat (Thermo Fisher Scientific, Waltham, MA, USA) and mounted on glass slides.

The sections were fixed in 4% paraformaldehyde (Servicebio, Wuhan, China) for 10 min at room temperature, rinsed with distilled water, and immersed in 60% isopropanol prepared from 2-propanol (ACS reagent, ≥99.5%; catalog no. 190764; Sigma-Aldrich, St. Louis, MO, USA). Subsequently, the sections were stained with freshly prepared Oil Red O working solution, consisting of 0.5% Oil Red O (catalog no. O0625; Sigma-Aldrich, St. Louis, MO, USA) in isopropanol diluted with distilled water at a ratio of 3:2, for 15 min at room temperature. Excess stain was removed by rinsing the sections with 60% isopropanol, followed by washing with distilled water. The sections were then counterstained with hematoxylin staining solution (catalog no. G1004; Servicebio, Wuhan, China) for 2 min, thoroughly rinsed with distilled water, and mounted using glycerin gelatin mounting medium (catalog no. G1402; Servicebio, Wuhan, China).

Stained sections were scanned using a digital slide scanner (Wisleap, Changzhou, China). Digital slides were examined using NDP.view2 software (version 2.9.22; Hamamatsu Photonics K.K., Hamamatsu, Japan), and five non-overlapping microscopic fields were selected from each muscle section for image quantification. Oil Red O staining was quantified using Fiji ImageJ software (ImageJ version 1.54f; National Institutes of Health, Bethesda, MD, USA). The integrated optical density (IOD) and Oil Red O-positive staining area were measured, and the mean optical density was calculated as the IOD divided by the positive staining area. The five field-level measurements were summarized as mean ± SD for each muscle for descriptive comparison.

### 2.3. Fatty Acid Extraction and Methylation

The extraction and methyl esterification procedures were performed according to the Chinese National Food Safety Standard GB 5009.168-2016 with minor modifications. Approximately 100 mg of homogenized muscle tissue was accurately weighed into a 2.0 mL microcentrifuge tube. Analytical-grade diethyl ether (Shanghai Lingfeng Chemical Reagent Co., Ltd., Shanghai, China) and a stainless-steel grinding bead were added, followed by homogenization, vortex mixing, and centrifugation. Each sample was extracted twice with diethyl ether, and the supernatants obtained from the two extractions were combined and evaporated to dryness under a stream of nitrogen.

The dried residue was saponified using NaOH–methanol solution prepared from sodium hydroxide (Guangzhou Chemical Reagent Factory, Guangzhou, China) and methanol (CNW; ANPEL Laboratory Technologies (Shanghai) Inc., Shanghai, China) in a water bath and subsequently methylated using 14% BF_3_–methanol solution (CNW; ANPEL Laboratory Technologies (Shanghai) Inc., Shanghai, China) to generate fatty acid methyl esters (FAMEs). Saturated sodium chloride solution, prepared from analytical-grade sodium chloride (Aladdin Biochemical Technology Co., Ltd., Shanghai, China), and chromatographic-grade *n*-hexane (CNW; ANPEL Laboratory Technologies (Shanghai) Inc., Shanghai, China) were added for phase separation. The mixture was vortexed at 1500 rpm for 3 min using an MS 3 digital vortex mixer (IKA, Staufen, Germany) and subsequently centrifuged at 500 rpm for 5 min at 4 °C using a 5427 R refrigerated centrifuge (Eppendorf, Hamburg, Germany). The upper *n*-hexane phase containing the FAMEs was collected and transferred into an injection vial for GC–MS/MS analysis.

For serum fatty acid analysis, 100 μL of serum was transferred into a microcentrifuge tube and subjected to the same saponification, methyl esterification, and phase-separation procedures described above for the muscle samples. Following vortex mixing at 1500 rpm for 3 min, the samples were centrifuged at 500 rpm for 5 min at 4 °C using a 5427 R refrigerated centrifuge (Eppendorf, Hamburg, Germany). The upper *n*-hexane phase was collected as described above and transferred into an injection vial for GC–MS/MS analysis.

### 2.4. Determination of Fatty Acids by GC–MS/MS

FAMEs were analyzed using a GC-2030 gas chromatograph coupled with a GCMS-TQ8040 NX triple-quadrupole mass spectrometer (Shimadzu Corporation, Kyoto, Japan), equipped with a TR-FAME capillary column (100 m × 0.25 mm i.d. × 0.20 μm film thickness; Thermo Fisher Scientific, Waltham, MA, USA).

A 1 μL aliquot of each sample was injected in split mode at a split ratio of 10:1, and the injector temperature was maintained at 250 °C. High-purity helium (99.999%) was used as the carrier gas at a constant flow rate of 1.0 mL/min.

The oven temperature program was as follows: the initial temperature was maintained at 120 °C for 5 min, increased to 175 °C at 10 °C/min and maintained for 10 min, increased to 210 °C at 5 °C/min and maintained for 5 min, and finally increased to 240 °C at 5 °C/min and maintained for 10 min.

Mass spectrometric detection was performed using electron ionization at 70 eV, and the ion source temperature was maintained at 230 °C. Data were acquired in multiple reaction monitoring (MRM) mode using optimized precursor-to-product ion transitions provided by LC-Bio Technology Co., Ltd. (Hangzhou, China).

Fatty acids were identified by comparing their retention times and MRM transitions with those obtained from a 37-component FAME reference mixture prepared using authentic FAME standards (purity > 99%; ANPEL Laboratory Technologies (Shanghai) Inc., Shanghai, China). No internal standard was used. Quantification was performed using analyte-specific external calibration curves prepared from serial dilutions of the FAME reference mixture. Fatty acid concentrations in muscle tissues were expressed as μg/g tissue, whereas serum fatty acid concentrations were expressed as μg/mL serum.

Quality-control samples prepared by pooling aliquots from all biological samples were analyzed periodically throughout the analytical sequence to monitor analytical stability and reproducibility.

### 2.5. Data Processing and Visualization

Fatty acid proportions were calculated separately for each sample by dividing the concentration of each fatty acid by the sum of all detected fatty acid concentrations and multiplying the resulting value by 100; non-detected values were assigned a value of zero for these calculations. Volcano plots were constructed using log_2_ fold changes and the corresponding unadjusted *p* values. Heatmaps and volcano plots were generated using the online analysis platform provided by LC-Bio Technology Co., Ltd. (Hangzhou, China), whereas the PLS-DA plots were generated in Python 3.12.13. Other graphs were generated using GraphPad Prism 9.5 (GraphPad Software Inc., San Diego, CA, USA), and the final figure layouts were assembled using Adobe Illustrator 2024 (Adobe Inc., San Jose, CA, USA).

### 2.6. Statistical Analysis

Unless otherwise specified, univariate statistical analyses were performed using GraphPad Prism 9.5. The nine animals constituted the independent experimental units, whereas the six anatomically defined muscles collected from each animal represented repeated within-animal observations. Differences in fatty acid concentrations among the six muscles were evaluated using linear mixed-effects models, with muscle type included as a fixed effect and animal identity as a random intercept to account for the correlation among measurements obtained from the same animal. Tukey’s multiple-comparisons test was subsequently used for pairwise comparisons among muscles. Individual fatty acid concentrations between the SS and SM muscles were compared using two-sided paired Student’s *t*-tests, with measurements paired by animal. A sensitivity analysis was conducted using G*Power version 3.1.9.7 (Heinrich Heine University Düsseldorf, Düsseldorf, Germany) by selecting “Sensitivity: Compute required effect size” for a two-tailed matched-pairs *t*-test. With a significance level of α = 0.05, statistical power of 1-β = 0.80, and a total sample size of nine paired observations, the minimum detectable effect size was Cohen’s *d*_z_ = 1.06697 (approximately 1.07). Overall variation in fatty acid profiles was further explored using a two-component binary PLS-DA model implemented in Python 3.12.13 with the NIPALS algorithm to distinguish SS from the other muscle and serum samples. Data were log_10_-transformed, mean-centered, and autoscaled before analysis. Model performance was evaluated using R^2^Y, leave-one-animal-out cross-validated Q^2^, and the area under the receiver operating characteristic curve (AUC), with significance assessed by 999 label permutations restricted within animal identity. The heatmap was generated using the LC-Bio online platform with the pheatmap package version 1.0.12 in R 3.6.3. Fatty acid concentrations were standardized by row-wise Z-score transformation, and the fatty acid columns were hierarchically clustered using Euclidean distance and complete linkage. Serum–muscle associations were assessed using Spearman’s rank correlation based on paired animal-level observations, and the resulting *p* values were adjusted using the Benjamini–Hochberg FDR procedure. Oil Red O IOD/area values obtained from five non-overlapping microscopic fields per muscle were analyzed using one-way analysis of variance followed by Tukey’s multiple-comparisons test. Unless otherwise stated, data are presented as mean ± standard deviation (SD). Statistical significance was defined as *p* < 0.05, whereas *q* < 0.05 was used for FDR-adjusted serum–muscle correlation analyses.

## 3. Results

### 3.1. Oil Red O Staining Patterns in Six Skeletal Muscles of Tianfu Goats

**Figure 1:**
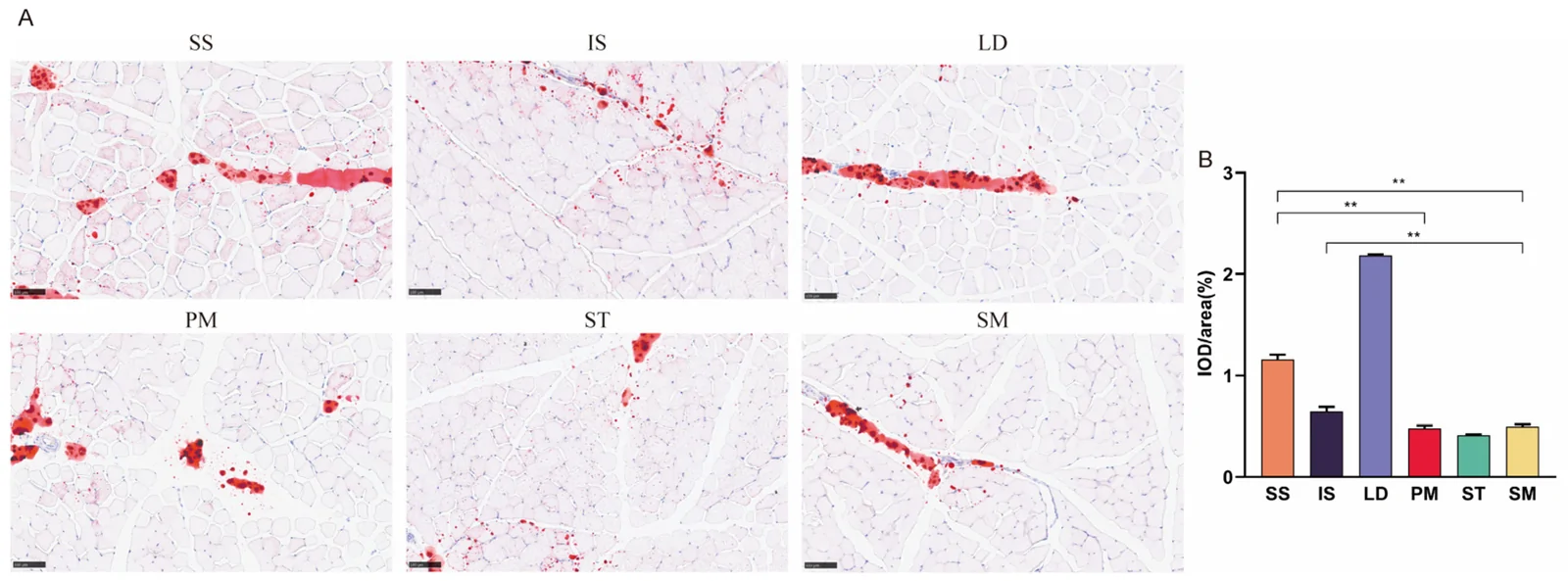
Oil Red O staining and semiquantitative image analysis of six skeletal muscles. (**A**) Oil Red O-stained cryosections of the *supraspinatus* (SS), *infraspinatus* (IS), *longissimus dorsi* (LD), *psoas major* (PM), *semitendinosus* (ST), and *semimembranosus* (SM) muscles. Red staining indicates Oil Red O-positive regions. Scale bar = 100 μm. (**B**) Semiquantitative comparison of Oil Red O-positive staining intensity among the six muscles based on integrated optical density per unit positive area (IOD/area). Data are presented as mean ± SD of five non-overlapping microscopic fields per muscle. Differences among muscles were evaluated using one-way analysis of variance followed by Tukey’s multiple-comparisons test. ** *p* < 0.01. IOD/area was used as a relative histological indicator of Oil Red O-positive staining intensity within the examined muscle sections.

Oil Red O staining revealed differences in lipid-associated staining patterns among the six skeletal muscles (Figure 1A). Oil Red O-positive signals were unevenly distributed across muscle sections and were mainly localized in the perimysial regions and between muscle fibers. Among the examined muscles, LD and SS exhibited stronger Oil Red O-positive staining intensity and larger Oil Red O-positive areas, whereas IS, PM, ST, and SM showed relatively weaker staining intensity and smaller positive areas.

Quantitative analysis based on the integrated optical density per unit area (IOD/area) of Oil Red O-positive staining (Figure 1B) revealed significant differences among muscles. LD exhibited the highest Oil Red O-associated staining intensity and was significantly higher than SS, IS, PM, ST, and SM (*p* < 0.01). SS also showed significantly higher Oil Red O-associated staining intensity than PM, ST, and SM (*p* < 0.01), while IS exhibited higher staining intensity than SM (*p* < 0.01). No significant differences were detected among PM, ST, and SM muscles (*p* > 0.05).

These results indicate that Oil Red O-positive staining intensity and distribution patterns varied among skeletal muscles, with the LD and SS muscles exhibiting relatively stronger staining signals than the other muscles examined. The IOD/area values provided a semiquantitative histological measure of the relative intensity of Oil Red O-positive staining within the examined sections and were used to compare lipid-associated staining levels and spatial distribution patterns among the six skeletal muscles.

### 3.2. Fatty Acid Composition and Proportions in Six Muscles of Tianfu Goats

**Figure 2:**
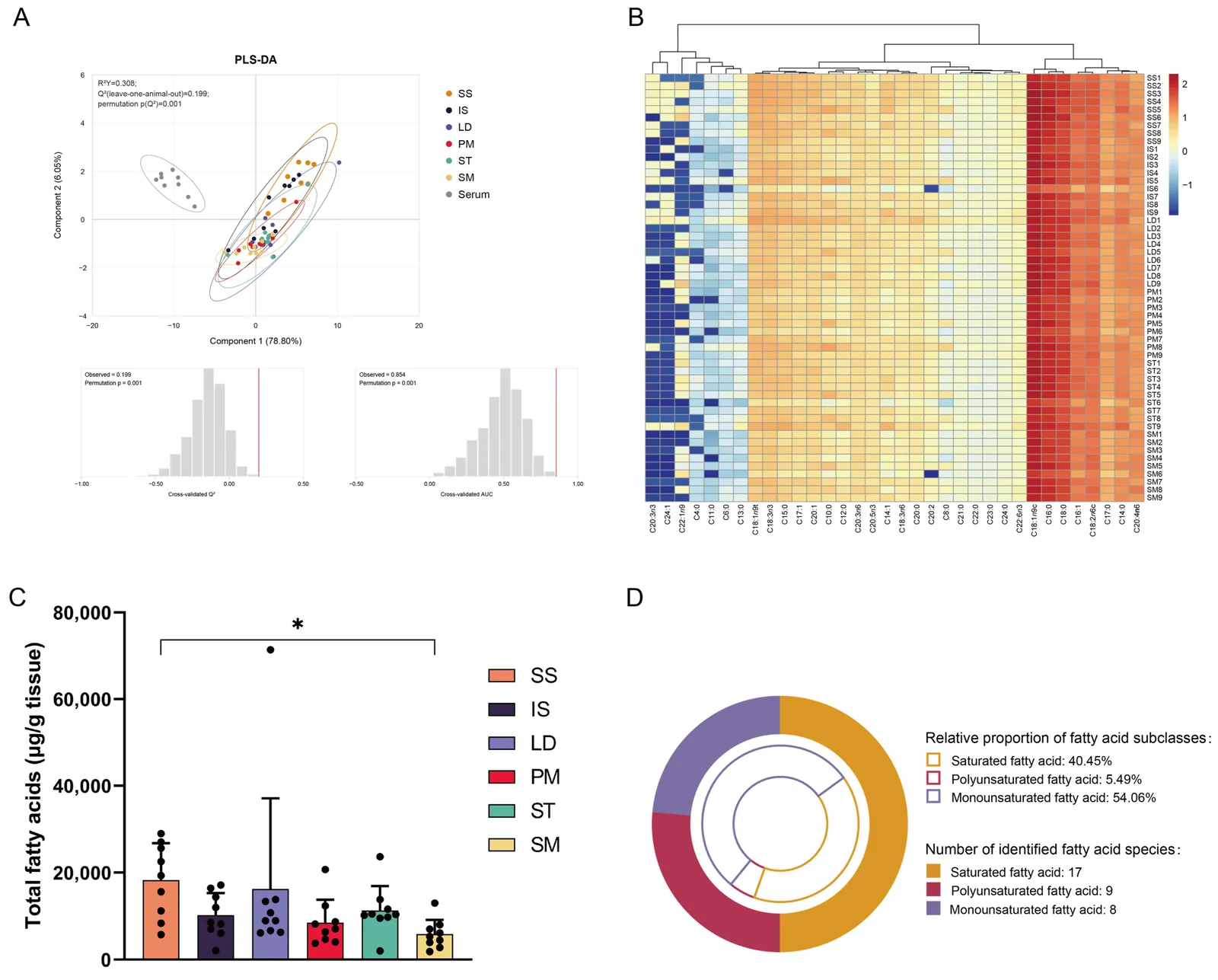
Comparative characterization of fatty acid profiles in six skeletal muscles and serum of Tianfu goats. (**A**) Partial least squares discriminant analysis (PLS-DA) score plot illustrating the distribution of serum and six skeletal muscle samples based on their fatty acid profiles. Each point represents an individual serum or muscle sample, with colors indicating sample types as shown in the legend. The corresponding colored ellipses illustrate within-group dispersion in the PLS-DA score plot. Model performance was evaluated using R^2^Y, Q^2^, and the area under the receiver operating characteristic curve (AUC) with leave-one-animal-out cross-validation. Statistical significance was assessed using 999 label permutations restricted within animal identity. In the permutation histograms, gray bars show the distributions of cross-validated Q^2^ and AUC values obtained from label permutations, and red vertical lines indicate the observed values. (**B**) Heatmap showing the row-wise Z-score-normalized relative abundance of 34 fatty acids across the six skeletal muscles. Hierarchical clustering was performed using Euclidean distance and complete linkage. Colors indicate the relative abundance of each fatty acid after row-wise Z-score normalization. (**C**) Total fatty acid concentrations in the six skeletal muscles. Data are presented as mean ± SD (*n* = 9; μg/g tissue). Bar colors identify the six muscle types as shown in the legend. Bars represent means, error bars indicate SD, and black dots represent individual animals. The horizontal bracket identifies the SS–SM comparison. Differences among muscle types were evaluated using a linear mixed-effects model, with muscle type as a fixed effect and animal identity as a random intercept, followed by Tukey’s multiple-comparisons test. * *p* < 0.05 indicates a significant difference between SS and SM. (**D**) Relative proportions of SFAs, MUFAs, and PUFAs in skeletal muscles, together with the number of fatty acid species identified in each subclass.

PLS-DA showed a clear separation between serum and skeletal muscle samples, whereas the six skeletal muscles displayed considerable overlap, indicating marked differences between serum and muscle fatty acid profiles but comparatively modest variation among muscles (Figure 2A). Across the six skeletal muscles, a total of 34 fatty acids were identified, of which C18:1*n*9c, C16:0, C18:0, and C18:2*n*6c were the predominant components; the concentrations of individual fatty acids in each muscle are presented in Table 2.

The Z-score-normalized heatmap revealed distinct distribution patterns of fatty acids among the different muscle samples (Figure 2B), consistent with the quantitative concentrations presented in Table 2. C18:1*n*9c was the most abundant fatty acid, with mean concentrations ranging from 2811.11 ± 1707.55 μg/g in SM to 9361.11 ± 4390.87 μg/g in SS. The concentrations of C16:0 ranged from 1364.00 ± 876.26 μg/g in SM to 3995.56 ± 1815.06 μg/g in SS, whereas C18:0 ranged from 823.00 ± 433.42 μg/g in SM to 2608.89 ± 4047.68 μg/g in LD. C18:2*n*6c concentrations ranged from 256.56 ± 82.56 μg/g in SM to 807.44 ± 370.95 μg/g in SS. In contrast, butyric acid (C4:0), caproic acid (C6:0), undecanoic acid (C11:0), tridecanoic acid (C13:0), 11,14,17-eicosatrienoic acid (C20:3*n*3), erucic acid (C22:1*n*9), and nervonic acid (C24:1) occurred at relatively low levels, with mean concentrations below 5.00 μg/g in all six muscles. C20:3*n*3 was not detected in SM, while C24:1 was not detected in PM or SM.

Among the six skeletal muscles, SS and LD showed relatively higher total fatty acid concentrations, whereas IS, PM, ST, and SM exhibited lower concentrations (Figure 2C). The total fatty acid concentration was significantly higher in SS than in SM based on the linear mixed-effects model (*p* < 0.05). The mean paired difference between SS and SM was 12,450.42 μg/g (95% CI: 5519.65–19,381.19), corresponding to Cohen’s *d*_z_ = 1.38 (95% CI: 0.43–2.29; Table S1). The 34 detected fatty acids comprised 17 SFAs, 8 MUFAs, and 9 PUFAs (Figure 2D). Based on the pooled mean concentrations across the six skeletal muscles, MUFAs accounted for the largest proportion of total fatty acids (54.06%), followed by SFAs (40.45%) and PUFAs (5.49%). Overall, Tianfu goat skeletal muscles exhibited a MUFA-dominant fatty acid composition together with muscle-dependent variation in total fatty acid abundance, characterized by relatively higher concentrations in SS and LD and a significant difference between SS and SM.

### 3.3. Fatty Acid Subclasses Varied Among Six Muscles of Tianfu Goats

**Figure 3:**
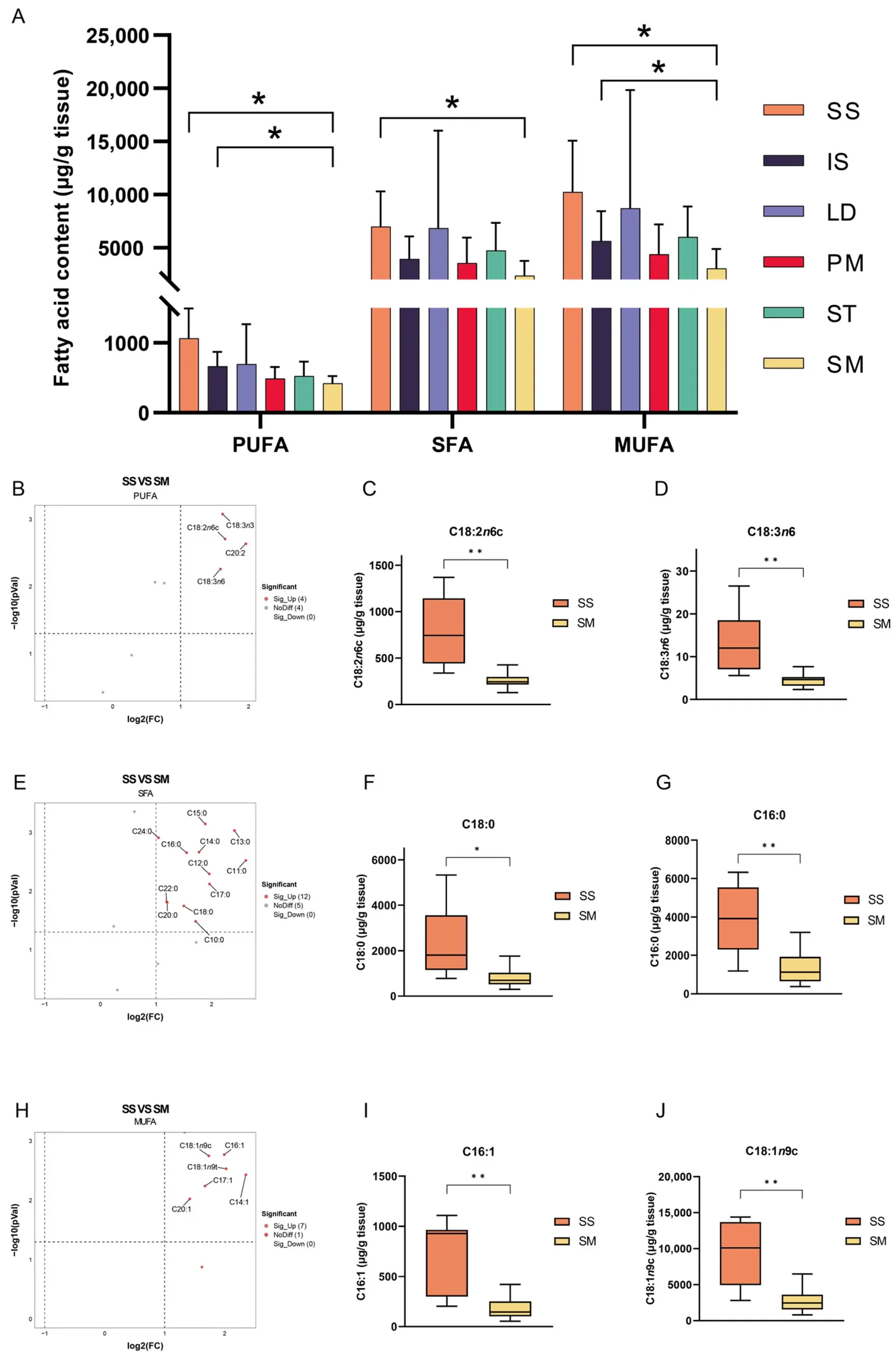
Differences in fatty acid subclasses and representative individual fatty acids among skeletal muscles. (**A**) Concentrations of polyunsaturated fatty acids (PUFAs), saturated fatty acids (SFAs), and monounsaturated fatty acids (MUFAs) across the six skeletal muscles. Differences among muscles were evaluated using a linear mixed-effects model, with muscle type as a fixed effect and animal identity as a random intercept, followed by Tukey’s multiple-comparisons test. (**B**,**E**,**H**) Volcano plots showing differences in PUFA, SFA, and MUFA profiles between the *supraspinatus* (SS) and *semimembranosus* (SM) muscles, respectively. Differential fatty acids were identified using the thresholds |log_2_(fold change)| > 1 and *p* < 0.05, with positive log_2_(fold change) values indicating higher concentrations in SS than in SM. The vertical dashed lines indicate log_2_(fold change) thresholds of −1 and 1, corresponding to fold changes of 0.5 and 2, respectively. The horizontal dashed line indicates the significance threshold of *p* = 0.05 (−log_10_(*p*) ≈ 1.30). (**C**,**D**) Comparisons of C18:2*n*6c and C18:3*n*6 concentrations between SS and SM. (**F**,**G**) Comparisons of C18:0 and C16:0 concentrations between SS and SM. (**I**,**J**) Comparisons of C16:1 and C18:1*n*9c concentrations between SS and SM. In panels (**C**,**D**,**F**,**G**,**I**,**J**), boxes span the 25th–75th percentiles, and horizontal lines within the boxes indicate the medians. Whiskers extend to the minimum and maximum values. Individual fatty acids between SS and SM were compared using two-sided paired Student’s *t*-tests. Fatty acid concentrations are expressed as μg/g tissue. In panel (**A**), bars represent means, and error bars indicate SD (*n* = 9). * *p* < 0.05; ** *p* < 0.01.

Comparison of fatty acid subclasses across the six skeletal muscles revealed clear muscle-dependent variation in PUFA, SFA, and MUFA concentrations (Figure 3A). SS contained 1065.99 ± 425.52 μg/g PUFAs, 6973.64 ± 3325.02 μg/g SFAs, and 10,242.46 ± 4812.55 μg/g MUFAs. The PUFA concentration in SS was significantly higher than those in PM, ST, and SM, exceeding the corresponding concentrations by more than twofold (*p* < 0.05). Similarly, the SFA and MUFA concentrations in SS were significantly higher than those in SM and were approximately threefold the corresponding values in SM (*p* < 0.05). These results identified SS as the muscle with the most consistently elevated concentrations of all three fatty acid subclasses, with the clearest differences observed between SS and SM. Paired SS–SM effect estimates for total SFAs, MUFAs, and PUFAs are provided in Table S1, with Cohen’s *d*_z_ values of 1.27, 1.44, and 1.47, respectively.

Based on these subclass-level differences, individual fatty acids were further compared between SS and SM. Among the PUFAs, four fatty acids showed significantly higher concentrations in SS than in SM (Figure 3B). C18:2*n*6c and γ-linolenic acid (C18:3*n*6) were selected for detailed comparison, with concentrations of 807.44 ± 370.95 and 13.30 ± 7.11 μg/g in SS, respectively, compared with 256.56 ± 82.56 and 4.43 ± 1.59 μg/g in SM. Both fatty acids were present at approximately threefold higher concentrations in SS (*p* < 0.01; Figure 3C,D). Among the SFAs, 12 fatty acids showed significantly higher concentrations in SS (Figure 3E). The concentrations of C18:0 and C16:0 were 2328.78 ± 1512.79 and 3995.56 ± 1815.06 μg/g in SS, respectively, compared with 823.00 ± 433.42 and 1364.00 ± 876.26 μg/g in SM, representing increases of more than twofold (*p* < 0.05 and *p* < 0.01, respectively; Figure 3F,G). Among the MUFAs, seven fatty acids were significantly more abundant in SS than in SM (Figure 3H). Palmitoleic acid (C16:1) and C18:1*n*9c reached 710.33 ± 353.00 and 9361.11 ± 4390.87 μg/g in SS, respectively, compared with 178.38 ± 113.01 and 2811.11 ± 1707.55 μg/g in SM, with both exceeding the corresponding concentrations in SM by more than threefold (*p* < 0.01; Figure 3I,J). The observed standardized differences between SS and SM were *d*_z_ = 1.46 for C18:2*n*6c, 1.22 for C18:3*n*6, 0.98 for C18:0, 1.30 for C16:0, 1.46 for C16:1, and 1.44 for C18:1*n*9c, with the corresponding mean paired differences and 95% CIs presented in Table S1. Overall, the SS–SM comparison revealed a broad elevation of individual PUFAs, SFAs, and MUFAs in SS, consistent with the subclass-level differences observed between the two muscles.

### 3.4. Serum Fatty Acid Composition Characteristics

**Figure 4:**
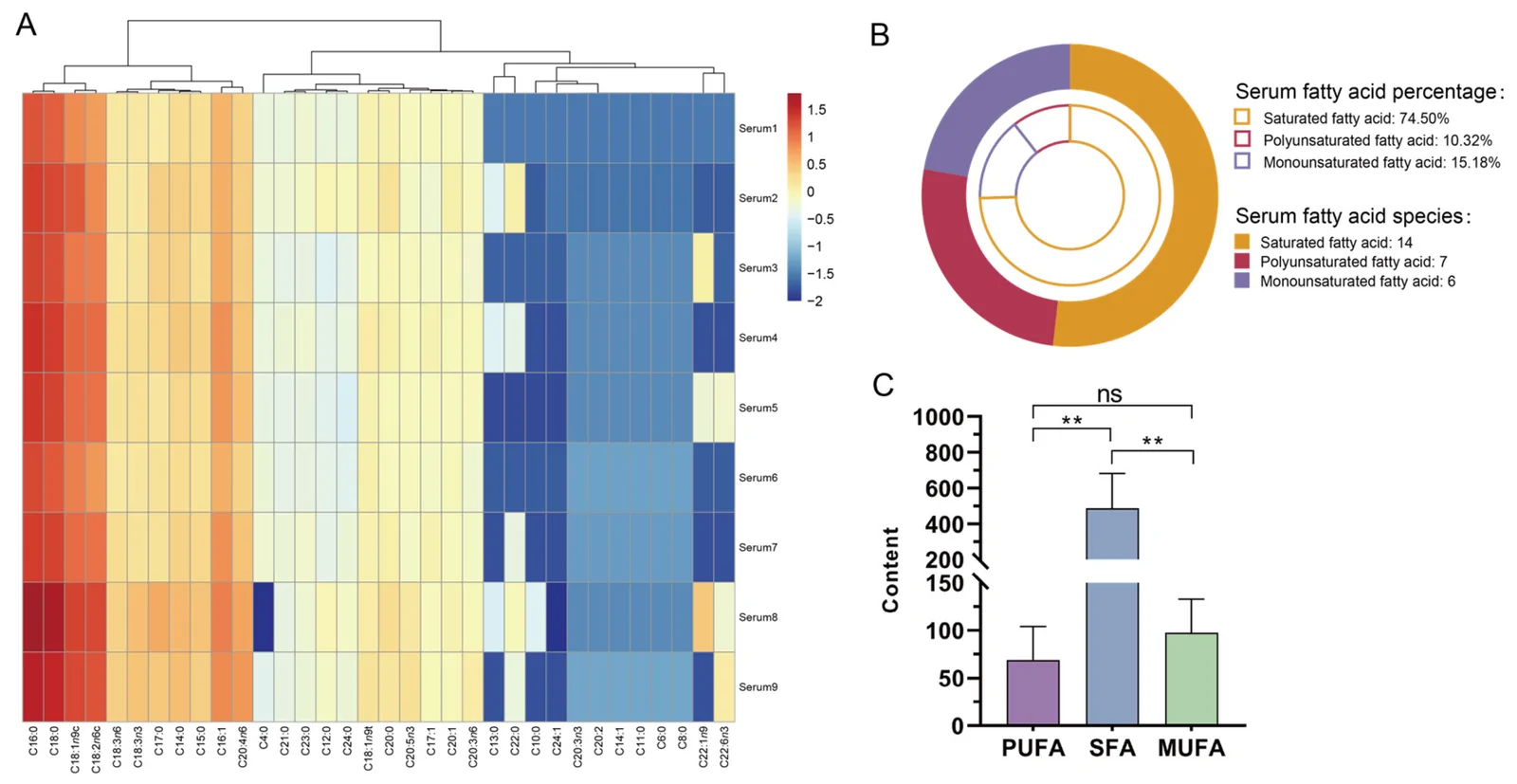
Serum fatty acid composition and comparison of fatty acid subclasses. (**A**) Heatmap showing Z-score normalized relative distribution patterns of 27 identified fatty acids in serum samples. The dendrogram above the heatmap represents hierarchical clustering of fatty acids based on their normalized profiles across serum samples. (**B**) Relative proportions of fatty acid subclasses, including SFAs, MUFAs, and PUFAs, in serum. (**C**) Concentrations of SFAs, MUFAs, and PUFAs in serum. Data are presented as mean ± SD and expressed as μg/mL serum. In panel (**C**), bars represent means, error bars indicate SD, and horizontal brackets indicate pairwise comparisons among fatty acid subclasses. Statistical comparisons among fatty acid subclasses were performed using repeated-measures one-way ANOVA followed by Tukey’s multiple-comparisons test. ns, not significant; ** *p* < 0.01.

A total of 27 fatty acids were detected and quantified in the serum samples, and their detailed concentrations and detection frequencies are presented in Table 3. The hierarchical clustering heatmap showed distinct distribution patterns among serum fatty acids based on their normalized relative abundance (Figure 4A). C16:0 was the most abundant serum fatty acid, with a concentration of 273.89 ± 112.21 μg/mL, followed by C18:0, C18:1*n*9c, and C18:2*n*6c, with concentrations of 197.11 ± 77.97, 74.60 ± 31.22, and 53.54 ± 26.74 μg/mL, respectively. In contrast, C13:0, heneicosanoic acid (C21:0), behenic acid (C22:0), and docosahexaenoic acid (C22:6*n*3) were detected at relatively low concentrations of 0.06 ± 0.09, 0.30 ± 0.07, 0.32 ± 0.43, and 0.26 ± 0.45 μg/mL, respectively.

The 27 detected fatty acids were classified into 14 SFAs, 6 MUFAs, and 7 PUFAs (Figure 4B). The total concentrations of SFAs, MUFAs, and PUFAs were 486.93 ± 195.34, 97.61 ± 35.29, and 68.97 ± 35.17 μg/mL, respectively (Table 3). Based on the proportions calculated separately for each animal, SFAs accounted for 74.50 ± 2.68% of total serum fatty acids, followed by MUFAs at 15.18 ± 2.23% and PUFAs at 10.32 ± 2.18% (Figure 4B). The concentration of SFAs was significantly higher than those of MUFAs and PUFAs (*p* < 0.01), whereas no significant difference was observed between MUFAs and PUFAs (*p* > 0.05; Figure 4C). These results demonstrate that the serum fatty acid profile of Tianfu goats was characterized by a predominance of SFAs.

### 3.5. Exploratory Analysis of Serum–Muscle Fatty Acid Associations

**Figure 5:**
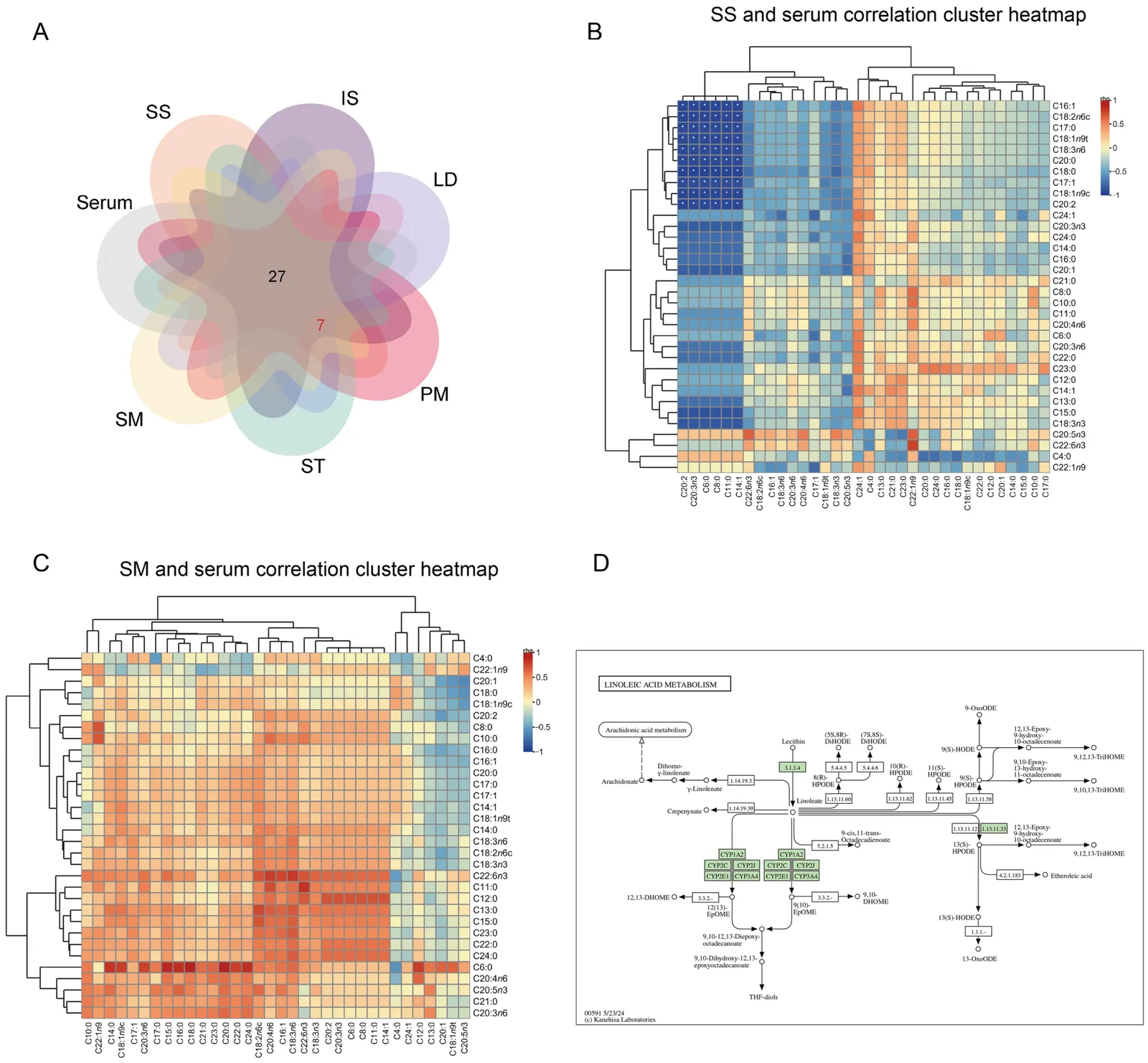
Exploratory analysis of serum–muscle fatty acid associations in Tianfu goats. (**A**) Venn diagram showing the overlap of identified fatty acid species among serum and six skeletal muscles (SS, IS, LD, PM, ST, and SM). Different colors represent the labeled tissues. The numbers 27 and 7 indicate fatty acid species shared by serum and skeletal muscle and those detected only in skeletal muscle, respectively. (**B**,**C**) Spearman correlation heatmaps showing the associations between fatty acid concentrations in serum and SS muscle (**B**) or SM muscle (**C**). Colors indicate Spearman’s correlation coefficients (ρ), ranging from blue for negative correlations through pale yellow for values near zero to red for positive correlations. The color bars show the coefficient scale from −1 to 1. The dendrograms above and to the left of the heatmaps show hierarchical clustering of the row and column fatty acids. Correlation coefficients were calculated based on individual animals (*n* = 9), and *p* values were adjusted using the Benjamini–Hochberg false discovery rate (FDR) correction. Asterisks indicate correlations that remained significant after FDR correction (* *q* < 0.05). (**D**) Mapping of identified fatty acids to the linoleic acid metabolism pathway based on the Kyoto Encyclopedia of Genes and Genomes (KEGG) database. Circles represent metabolites, and rectangular boxes represent enzymes or their corresponding genes. Green boxes indicate enzyme-associated genes annotated in the goat KEGG map, while white boxes show other enzyme entries. Boxed numeric labels are Enzyme Commission (EC) numbers, and CYP labels denote cytochrome P450 enzymes. Solid connecting lines and arrows indicate metabolic reactions and their directions. The dashed arrow links this map to the arachidonic acid metabolism pathway. This panel represents pathway mapping of detected fatty acids rather than statistical pathway enrichment analysis.

A total of 34 fatty acids were detected across the six skeletal muscles, of which 27 were also detected in serum. Seven fatty acids, including C6:0, caprylic acid (C8:0), C11:0, myristoleic acid (C14:1), eicosadienoic acid (C20:2), C20:3*n*3, and C24:1, were detected only in skeletal muscle, whereas no fatty acid was detected exclusively in serum (Figure 5A and Table 4). Thus, serum and skeletal muscle shared most of the detected fatty acid species, while skeletal muscle contained several additional fatty acids. Among the detected PUFAs, C18:2*n*6c, C18:3*n*6, C20:3*n*6, and C20:4*n*6 were represented within the linoleic acid metabolism pathway (Figure 5D), placing the observed *n*-6 PUFA profile within a connected metabolic framework.

Spearman correlation analyses with false discovery rate correction were subsequently performed to evaluate the associations between serum and muscle fatty acid profiles. Because SS and SM exhibited distinct fatty acid compositions, these two muscles were selected for detailed visualization (Figure 5B,C). The SS–serum correlation matrix displayed a heterogeneous pattern, with both positive and negative associations among individual fatty acids (Figure 5B). In contrast, SM showed a more consistently positive association pattern, and several major fatty acids, including C16:0, C18:0, C18:1*n*9c, and C18:2*n*6c, exhibited positive associations between serum and muscle concentrations (Figure 5C). Overall, the distinct correlation patterns observed for SS and SM indicate that the relationships between circulating and intramuscular fatty acid profiles differ among anatomical muscles.

## 4. Discussion

Skeletal muscles differ in their physiological functions and lipid-associated characteristics, which can lead to anatomical variation in meat composition. In the present study, Oil Red O staining provided a histological overview of this variation, with LD and SS showing relatively stronger positive staining than the other muscles examined. Similar intermuscular heterogeneity has been reported in small ruminants. Saengsuk et al. observed differences in fat content, lipid classes, and fatty acid composition among goat primal cuts, while Xu et al. identified distinct muscle fiber characteristics, fatty acid profiles, and lipidomic patterns among different muscles of Chaka sheep [8,25]. Anderson et al. also reported greater intramuscular fat deposition in SS than in SM in lambs [12], which is consistent with the stronger Oil Red O-positive staining observed in SS in the present study. These findings support the view that anatomical muscle location is an important source of variation in lipid-associated histological characteristics.

Consistent with the histological observations, the six skeletal muscles shared a broadly similar core fatty acid profile but differed in total fatty acid abundance and fatty acid subclass concentrations. SS and LD generally exhibited higher total fatty acid concentrations, whereas SM showed comparatively lower concentrations. SS was particularly distinct, as its PUFA, SFA, and MUFA concentrations were all higher than those in SM. The considerable overlap among the six muscles in the PLS-DA score plot further indicated that the intermuscular differences mainly reflected variation in the abundance of selected fatty acids and subclasses rather than completely distinct fatty acid profiles. Similar muscle-specific differences have been reported in other small ruminants. Hwang et al. found that fatty acid composition differed among the *longissimus lumborum*, PM, SM, and GM muscles of Korean native black goats [14]. In addition, Leng et al. showed that variation in fatty acid composition in goats and sheep was more strongly associated with anatomical muscle region than with species [26]. These findings are consistent with the present results and further demonstrate that fatty acid composition is muscle dependent, supporting the evaluation of multiple anatomical muscles rather than relying on a single muscle to represent the fatty acid characteristics of the entire carcass.

At the individual fatty acid level, C18:1*n*9c, C16:0, C18:0, and C18:2*n*6c were the predominant components of Tianfu goat skeletal muscles, and MUFAs constituted the largest fatty acid subclass. This compositional pattern is broadly consistent with previous studies of small ruminants. Saengsuk et al. identified C18:1*n*9, C16:0, and C18:0 as the principal fatty acids across the shoulder, rib, loin, breast, and leg cuts of goats [8]. Similarly, Leng et al. reported that oleic-, palmitic-, and stearic-acid derivatives were abundant across multiple skeletal muscles in both goats and sheep [26]. Studies of Korean native black goats also showed that C18:1, C16:0, and C18:0 were the major fatty acids in different anatomical muscles, although their relative proportions varied among muscles [14]. In Chaka sheep, C18:2*n*6c was likewise identified as a major PUFA, accompanied by muscle-dependent differences in fatty acid and lipid-class composition [25]. Thus, the major fatty acid profile observed in Tianfu goats follows a relatively conserved pattern in small-ruminant meat, whereas the abundance and distribution of individual fatty acids remain muscle dependent [25,27]. The predominance of MUFAs was mainly attributable to the abundance of oleic acid. Controlled dietary studies have shown that replacing palmitic acid with oleic acid may reduce low-density lipoprotein cholesterol and non-high-density lipoprotein cholesterol [28,29], and studies involving beef relatively enriched in oleic acid have reported neutral or potentially favorable effects on circulating lipoprotein profiles [30,31]. Linoleic acid is also nutritionally important because it is an essential *n*-6 PUFA that must be obtained from the diet, and replacing part of dietary SFA intake with PUFAs has been associated with more favorable cardiovascular outcomes [32,33,34,35]. The relatively high abundance of oleic acid and linoleic acid in SS therefore suggests that this muscle has considerable potential for nutrition-oriented product differentiation and value-added utilization. Nevertheless, its nutritional characteristics should be interpreted from the overall fatty acid profile because SS also contained relatively high total fatty acid and SFA concentrations.

Serum fatty acid profiling provided a complementary reference for interpreting the muscle results. The principal fatty acids detected in serum, including C16:0, C18:0, C18:1*n*9c, and C18:2*n*6c, were also major components of skeletal muscle, consistent with previous findings in goats [15]. However, SFAs predominated in serum, whereas MUFAs constituted the largest subclass in skeletal muscle, reflecting differences between circulating lipid pools and fatty acids incorporated into storage and structural lipids within muscle [36,37,38]. The different serum–muscle correlation patterns observed for SS and SM further suggest that the association between circulating and intramuscular fatty acid profiles may vary among anatomical muscles. Thus, serum analysis provided supporting physiological context for the observed intermuscular differences without altering the primary focus on muscle fatty acid composition.

Several considerations should be taken into account when interpreting the present findings. Given the exploratory design and the inclusion of nine independent animals, the sensitivity analysis indicated that individual two-sided paired comparisons were primarily sensitive to large effects (minimum detectable Cohen’s *d*_z_ = 1.07). Small or moderate effects may therefore have gone undetected, and non-significant findings should not be interpreted as evidence of equivalence or the absence of an effect. Oil Red O staining was used as a semiquantitative histological indicator of lipid-associated staining rather than as a direct chemical measurement of intramuscular fat content. Individual voluntary feed intake, energy retention, and dietary fatty acid composition were not measured, while odor-active compounds and sensory attributes were beyond the scope of the study. These limitations restrict assessment of nutritional contributions to the observed fatty acid profiles and their potential flavor implications. The paired within-animal design and targeted fatty acid quantification nevertheless provided a basis for characterizing intermuscular variation within this cohort under the reported farm feeding and management regimen. Further studies incorporating individual feed-intake measurements and larger independent cohorts would help clarify nutritional influences and validate the exploratory serum–muscle associations.

Overall, Tianfu goat skeletal muscles exhibited a shared MUFA-dominant fatty acid profile but differed in Oil Red O-positive staining, total fatty acid abundance, fatty acid subclass concentrations, and the distribution of nutritionally relevant individual fatty acids. SS showed the most pronounced enrichment of several MUFAs and PUFAs, particularly oleic and linoleic acids, indicating considerable potential for nutritional differentiation and value-added utilization. LD also exhibited relatively high lipid-associated characteristics, whereas SM generally contained lower fatty acid concentrations. The major fatty acids shared by serum and muscle, together with the muscle-dependent serum–muscle association patterns, provided additional physiological context for these anatomical differences. These findings provide a compositional basis for the nutritional evaluation, muscle-specific utilization, and future product development of Tianfu goat meat.

## 5. Conclusions

This study revealed clear anatomical variation in the fatty acid composition of Tianfu goat skeletal muscles. Thirty-four fatty acids were detected in muscle, with C18:1*n*9c, C16:0, C18:0, and C18:2*n*6c as the predominant components and MUFAs as the largest subclass. SS and LD exhibited relatively high lipid-associated characteristics, while SS contained higher total fatty acid and fatty acid subclass concentrations than SM, including greater amounts of nutritionally relevant oleic and linoleic acids. Serum shared most major fatty acids with skeletal muscle and provided complementary evidence of muscle-specific association patterns. Within the context of this exploratory design, small or moderate effects may have gone undetected in comparisons that did not reach statistical significance; nevertheless, the principal SS–SM comparisons showed consistently large observed effects. Together, these findings highlight SS as a muscle with considerable potential for nutritional differentiation and provide a basis for the muscle-specific evaluation and value-added utilization of Tianfu goat meat.

## Figures and Tables

**Table 1:** Nutrient composition of feed ingredients offered to Tianfu goats.

Nutrient Component	Oat Hay	Broad Bean Hulls	Concentrate
DM (%)	90	90	≥86
CP (%)	10	13	≥18
CF (%)	25	28	≤12
ADF (%)	39	46	—
NDF (%)	63	62	—
Crude fat (EE, %)	2.3	2.6	≥2.5
Ash (%)	8	5	≤10
Ca (%)	0.4	0.55	0.5–2.0
P (%)	0.27	0.17	≤0.4

Nutrient values for oat hay and broad bean hulls were determined by chemical analysis, whereas those for the concentrate were based on the manufacturer’s guaranteed composition. Crude fat in the concentrate represents the manufacturer-declared value, while fat contents of oat hay and broad bean hulls were determined as ether extract. No supplemental lipid source was added to the ration. DM, dry matter; CP, crude protein; CF, crude fiber; ADF, acid detergent fiber; NDF, neutral detergent fiber; EE, ether extract; Ca, calcium; P, phosphorus.

**Table 2:** Fatty acid concentrations in six skeletal muscles of Tianfu goats.

Class	Fatty Acid	SS	IS	LD	PM	ST	SM
SFA	C4:0	0.27 ± 0.21	0.15 ± 0.22	0.18 ± 0.18	0.14 ± 0.13	0.28 ± 0.24	0.22 ± 0.13
SFA	C6:0	0.66 ± 0.66	0.25 ± 0.30	0.51 ± 0.67	0.38 ± 0.37	0.33 ± 0.21	0.32 ± 0.20
SFA	C8:0	4.47 ± 4.51	3.13 ± 4.92	3.77 ± 4.59	3.68 ± 6.53	2.49 ± 2.87	1.35 ± 1.08
SFA	C10:0	29.14 ± 23.23	20.45 ± 27.57	25.84 ± 30.67	21.43 ± 34.72	19.09 ± 17.63	8.89 ± 7.18
SFA	C11:0	0.42 ± 0.25	0.24 ± 0.27	0.23 ± 0.35	0.16 ± 0.28	0.24 ± 0.21	0.06 ± 0.05
SFA	C12:0	19.73 ± 11.58	12.69 ± 15.75	13.30 ± 18.30	8.88 ± 10.82	14.62 ± 16.51	5.08 ± 3.02
SFA	C13:0	1.48 ± 0.72	0.84 ± 0.66	0.99 ± 1.68	0.48 ± 0.49	0.96 ± 1.03	0.28 ± 0.15
SFA	C14:0	360.11 ± 175.55	216.03 ± 142.29	299.67 ± 395.90	147.17 ± 109.35	259.32 ± 213.43	105.37 ± 57.14
MUFA	C14:1	24.43 ± 14.74	12.33 ± 10.77	10.50 ± 12.01	5.29 ± 3.68	16.49 ± 19.94	4.78 ± 4.29
SFA	C15:0	45.70 ± 19.50	28.21 ± 16.90	36.75 ± 57.28	18.67 ± 13.61	34.89 ± 29.88	12.37 ± 6.33
SFA	C16:0	3995.56 ± 1815.06	2313.00 ± 1376.06	3691.11 ± 4353.81	2008.33 ± 1443.10	2796.00 ± 1525.09	1364.00 ± 876.26
MUFA	C16:1	710.33 ± 353.00	372.69 ± 208.79	461.22 ± 602.69	244.44 ± 141.29	412.79 ± 268.15	178.38 ± 113.01
SFA	C17:0	165.87 ± 104.67	90.64 ± 48.10	150.36 ± 259.63	73.37 ± 61.13	103.32 ± 64.77	42.56 ± 21.73
MUFA	C17:1	34.56 ± 19.16	19.51 ± 9.60	29.81 ± 44.10	13.43 ± 8.20	19.07 ± 9.92	10.84 ± 6.09
SFA	C18:0	2328.78 ± 1512.79	1231.44 ± 549.75	2608.89 ± 4047.68	1263.22 ± 731.44	1482.11 ± 828.85	823.00 ± 433.42
MUFA	C18:1*n*9t	73.81 ± 40.15	36.37 ± 19.32	51.09 ± 74.92	26.31 ± 16.27	36.26 ± 24.00	18.13 ± 10.70
MUFA	C18:1*n*9c	9361.11 ± 4390.87	5157.89 ± 2575.32	8113.33 ± 10,369.62	4066.67 ± 2644.49	5512.22 ± 2547.19	2811.11 ± 1707.55
PUFA	C18:2*n*6c	807.44 ± 370.95	458.44 ± 162.37	476.22 ± 469.28	326.11 ± 129.79	350.89 ± 163.21	256.56 ± 82.56
PUFA	C18:3*n*6	13.30 ± 7.11	7.68 ± 2.76	7.98 ± 7.98	5.53 ± 2.43	5.97 ± 3.05	4.43 ± 1.59
PUFA	C18:3*n*3	64.39 ± 26.10	38.94 ± 16.89	43.32 ± 45.90	28.66 ± 15.93	34.97 ± 20.48	20.98 ± 9.73
SFA	C20:0	12.77 ± 7.03	7.89 ± 3.25	15.06 ± 25.17	7.80 ± 4.28	7.89 ± 5.52	5.55 ± 2.25
MUFA	C20:1	32.69 ± 18.12	16.54 ± 7.24	25.19 ± 28.23	16.34 ± 12.06	19.62 ± 7.69	12.23 ± 5.04
SFA	C21:0	1.67 ± 0.30	1.52 ± 0.15	1.77 ± 1.07	1.48 ± 0.20	1.50 ± 0.31	1.41 ± 0.14
PUFA	C20:2	10.86 ± 5.58	4.90 ± 3.14	6.10 ± 6.47	3.89 ± 2.75	4.43 ± 2.51	2.72 ± 1.35
PUFA	C20:3*n*6	17.10 ± 5.21	12.82 ± 3.19	15.67 ± 6.39	10.92 ± 2.57	12.10 ± 2.85	11.10 ± 1.82
PUFA	C20:4*n*6	139.42 ± 36.77	128.28 ± 23.95	134.78 ± 31.78	103.09 ± 16.79	103.59 ± 20.60	114.88 ± 20.60
PUFA	C20:3*n*3	2.32 ± 1.48	1.02 ± 1.03	0.69 ± 1.75	0.28 ± 0.85	0.31 ± 0.93	ND
SFA	C22:0	2.32 ± 1.27	1.62 ± 0.46	1.91 ± 1.91	1.25 ± 0.42	1.22 ± 0.56	1.02 ± 0.24
MUFA	C22:1*n*9	4.22 ± 5.55	0.07 ± 0.21	3.55 ± 5.85	1.68 ± 1.71	3.59 ± 4.60	1.18 ± 1.71
PUFA	C20:5*n*3	7.29 ± 1.90	7.92 ± 1.87	9.65 ± 2.49	7.38 ± 1.48	7.99 ± 1.74	8.06 ± 1.63
SFA	C23:0	1.55 ± 0.30	1.40 ± 0.24	1.33 ± 0.83	1.04 ± 0.19	0.94 ± 0.20	1.02 ± 0.16
SFA	C24:0	3.14 ± 1.02	2.44 ± 0.47	2.37 ± 1.42	1.57 ± 0.38	1.59 ± 0.47	1.52 ± 0.28
MUFA	C24:1	1.31 ± 1.31	1.27 ± 0.96	0.79 ± 1.67	ND	0.24 ± 0.73	ND
PUFA	C22:6*n*3	3.86 ± 1.38	3.02 ± 1.12	3.61 ± 2.73	2.41 ± 0.92	3.18 ± 1.58	2.28 ± 0.55
Summary	Total SFAs	6973.64 ± 3325.02	3931.96 ± 2135.41	6854.04 ± 9170.00	3559.05 ± 2395.56	4726.79 ± 2622.27	2374.02 ± 1383.12
Summary	Total MUFAs	10,242.46 ± 4812.55	5616.67 ± 2820.87	8695.48 ± 11,129.69	4374.16 ± 2812.63	6020.29 ± 2852.42	3036.65 ± 1841.48
Summary	Total PUFAs	1065.99 ± 425.52	663.03 ± 207.51	698.03 ± 571.54	488.27 ± 167.65	523.42 ± 209.67	421.00 ± 103.96
Summary	Total fatty acids	18,282.09 ± 8516.92	10,211.66 ± 5098.68	16,247.55 ± 20,866.96	8421.47 ± 5345.72	11,270.50 ± 5645.36	5831.66 ± 3310.31

Values are expressed as μg/g tissue and presented as mean ± SD for nine animals (*n* = 9), with one biological observation per animal for each muscle. ND indicates that a fatty acid was not detected in any sample from the corresponding muscle. ND values were treated as zero when calculating mean concentrations, fatty acid subclass totals, and total fatty acid concentrations. Subclass and total fatty acid concentrations were calculated for each animal before group-level summarization. cis-10-pentadecenoic acid (C15:1), linolelaidic acid (C18:2*n*6t), and cis-13,16-docosadienoic acid (C22:2) were not detected in any muscle and are therefore omitted. SS, *supraspinatus*; IS, *infraspinatus*; LD, *longissimus dorsi*; PM, *psoas major*; ST, *semitendinosus*; SM, *semimembranosus*; SFA, saturated fatty acid; MUFA, monounsaturated fatty acid; PUFA, polyunsaturated fatty acid; ND, not detected.

**Table 3:** Serum fatty acid concentrations and detection frequencies in Tianfu goats.

Class	Fatty Acid	Concentration (μg/mL Serum)	Detection Frequency
SFA	C4:0	0.29 ± 0.13	8/9
SFA	C10:0	0.02 ± 0.07	1/9
SFA	C12:0	0.41 ± 0.30	9/9
SFA	C13:0	0.06 ± 0.09	3/9
SFA	C14:0	4.79 ± 1.53	9/9
SFA	C15:0	3.56 ± 0.89	9/9
SFA	C16:0	273.89 ± 112.21	9/9
SFA	C17:0	3.99 ± 2.56	9/9
SFA	C18:0	197.11 ± 77.97	9/9
SFA	C20:0	1.43 ± 0.72	9/9
SFA	C21:0	0.30 ± 0.07	9/9
SFA	C22:0	0.32 ± 0.43	5/9
SFA	C23:0	0.40 ± 0.10	9/9
SFA	C24:0	0.36 ± 0.20	9/9
MUFA	C16:1	19.59 ± 5.92	9/9
MUFA	C17:1	0.74 ± 0.23	9/9
MUFA	C18:1*n*9t	1.23 ± 0.44	9/9
MUFA	C18:1*n*9c	74.60 ± 31.22	9/9
MUFA	C20:1	0.81 ± 0.18	9/9
MUFA	C22:1*n*9	0.64 ± 1.40	3/9
PUFA	C18:2*n*6c	53.54 ± 26.74	9/9
PUFA	C18:3*n*6	2.57 ± 1.00	9/9
PUFA	C18:3*n*3	2.99 ± 1.72	9/9
PUFA	C20:3*n*6	0.72 ± 0.30	9/9
PUFA	C20:4*n*6	7.91 ± 5.05	9/9
PUFA	C20:5*n*3	0.97 ± 0.68	9/9
PUFA	C22:6*n*3	0.26 ± 0.45	3/9
Summary	Total SFAs	486.93 ± 195.34	—
Summary	Total MUFAs	97.61 ± 35.29	—
Summary	Total PUFAs	68.97 ± 35.17	—
Summary	Total fatty acids	653.51 ± 258.47	—

Values are presented as mean ± SD across nine animals (*n* = 9). Non-detected values were assigned zero for descriptive calculations. Detection frequency indicates the number of serum samples in which each fatty acid was detected. C6:0, C8:0, C11:0, C14:1, C20:2, C20:3*n*3, and C24:1 were not detected in any serum sample. SFA, saturated fatty acid; MUFA, monounsaturated fatty acid; PUFA, polyunsaturated fatty acid.

**Table 4:** Distribution of fatty acid species detected in skeletal muscle and serum of Tianfu goats.

Detection Category	Class	Fatty Acids
Muscle and serum	SFA	C4:0, C10:0, C12:0, C13:0, C14:0, C15:0, C16:0, C17:0, C18:0, C20:0, C21:0, C22:0, C23:0, C24:0
	MUFA	C16:1, C17:1, C18:1*n*9t, C18:1*n*9c, C20:1, C22:1*n*9
	PUFA	C18:2*n*6c, C18:3*n*6, C18:3*n*3, C20:3*n*6, C20:4*n*6, C20:5*n*3, C22:6*n*3
Muscle only	SFA	C6:0, C8:0, C11:0
	MUFA	C14:1, C24:1
	PUFA	C20:2, C20:3*n*3

Detection indicates that the fatty acid was detected in at least one sample of the corresponding biological matrix. SFA, saturated fatty acid; MUFA, monounsaturated fatty acid; PUFA, polyunsaturated fatty acid.

## Data Availability

The original contributions presented in this study are included in the article/Supplementary Material. Further inquiries can be directed to the corresponding authors.

## Supplementary Materials

The following supporting information can be downloaded at: https://www.mdpi.com/article/10.3390/foods15203586/s1, Table S1: Paired effect estimates for the principal fatty acid comparisons between the *supraspinatus* and *semimembranosus* muscles.

## Abbreviations

The following abbreviations are used in this manuscript:

SS	*supraspinatus*
SM	*semimembranosus*
MUFA	monounsaturated fatty acid
SFA	saturated fatty acid
PUFA	polyunsaturated fatty acid
IS	*infraspinatus*
LD	*longissimus dorsi*
PM	*psoas major*
ST	*semitendinosus*
OCT	optimal cutting temperature compound
FAMEs	fatty acid methyl esters
MRM	multiple reaction monitoring
FDR	false discovery rate
IOD	integrated optical density
PLS-DA	partial least squares discriminant analysis
GC–MS/MS	gas chromatography–tandem mass spectrometry
AUC	area under the receiver operating characteristic curve
KEGG	Kyoto Encyclopedia of Genes and Genomes
GM	*gluteus medius*
