# Supplementary material for: Comparative Analysis of Fatty Acid Composition in Skeletal Muscles and Serum of Tianfu Goats
Source: Foods. 2026 Oct 9;15(20):3586. doi: 10.3390/foods15203586 (PMC13703456; doi:10.3390/foods15203586)
Supplement: Supplementary file 1 [file foods-15-03586-s001.zip › foods-4578567-supplementary.pdf]

Supplementary Materials

Table S1. Paired effect estimates for the principal fatty acid comparisons between the *supraspinatus* and *semimembranosus* muscles.

Comparison	Mean paired difference, SS - SM (μg/g)	95% CI of difference	Cohen's d_z	95% CI of d_z
Total fatty acids	12,450.42	5,519.65 to 19,381.19	1.38	0.43 to 2.29
Total SFAs	4,599.62	1,805.91 to 7,393.32	1.27	0.35 to 2.14
Total MUFAs	7,205.81	3,370.93 to 11,040.69	1.44	0.47 to 2.38
Total PUFAs	645.00	307.93 to 982.07	1.47	0.49 to 2.41
C18:2*n*6c	550.89	261.12 to 840.66	1.46	0.48 to 2.40
C18:3*n*6	8.88	3.28 to 14.48	1.22	0.32 to 2.08
C18:0	1,505.78	321.53 to 2,690.03	0.98	0.15 to 1.76
C16:0	2,631.56	1,071.74 to 4,191.37	1.30	0.37 to 2.18
C16:1	531.96	251.03 to 812.88	1.46	0.48 to 2.39
C18:1*n*9c	6,550.00	3,050.56 to 10,049.44	1.44	0.47 to 2.37

Values are based on paired observations from nine animals. Mean differences were calculated as SS - SM. Cohen's d_z was calculated as the mean within-animal difference divided by the SD of the paired differences; 95% CIs for d_z were derived using the noncentral t distribution. For total fatty acids and fatty acid subclasses, statistical significance was determined using linear mixed-effects models followed by Tukey's multiple-comparisons test. For the six selected individual fatty acids, statistical significance was determined using two-sided paired Student's t-tests. Positive values indicate higher concentrations in SS than in SM. SFA, saturated fatty acid; MUFA, monounsaturated fatty acid; PUFA, polyunsaturated fatty acid; CI, confidence interval.
